# Pyruvate Carboxylase as a Moonlighting Metabolic Enzyme Protects β‐Cell From Senescence

**DOI:** 10.1111/1753-0407.70050

**Published:** 2025-02-13

**Authors:** Yumei Yang, Baomin Wang, Xiaomu Li

**Affiliations:** ^1^ Department of Endocrinology and Metabolism, Zhongshan Hospital Fudan University Shanghai China

**Keywords:** diabetes, pyruvate carboxylase, senescence

## Abstract

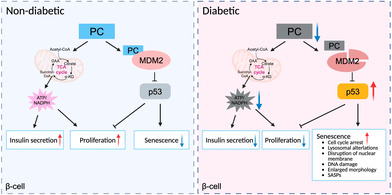

**Summary**

- PC is essential for maintaining healthy β‐cell functions in diabetic and aging conditions. PC expression was downregulated under diabetic and aging conditions. Replenishment of PC alleviated defective GSIS and senescence in islets.
- PC interacts with MDM2 via a binding motif at the C‐terminus of PC, which prevented MDM2 proteasomal degradation and hence increased its expression, thereby limiting p53‐dependent β‐cell senescence.
- PC involves in glucose‐stimulated insulin secretion (GSIS) via the MDM2–p53–PC axis and plays anti‐senescence function via the PC–MDM2–p53 axis, highlighting the vital role of PC in diabetes therapy and/or prevention.

Pyruvate carboxylase (PC), a mitochondrial anaplerotic enzyme, plays a critical role in various pancreatic β‐cell pathways, including insulin secretion [1], proliferation [2], inflammation [3], and apoptosis [4]. Down‐regulation of PC expression in pancreatic β‐cells/islets has been known to be associated with diabetes in humans and rodents [5, 6]. However, the molecular mechanisms remain elusive. Recently, a study published by our group in *the Proceedings of the National Academy of Sciences* (*PNAS*) demonstrated that PC involves in an anti‐senescence program in β‐cells, providing a new insight into the relationship between PC and glucose metabolism [7]. Herein, we would like to provide critical commentary on this article entitled “The mitochondrial enzyme pyruvate carboxylase restricts pancreatic β‐cell senescence by blocking p53 activation.”

While PC is known to control insulin secretion by generating coupling factors ATP and NADPH in β‐cell lines or isolated islets [1], its physiological function in vivo has never been fully explored. To address this research gap, the researchers generated β‐cell specific PC knockout (β‐PCKO) mice to determine the physiological functions of PC in pancreatic β‐cells. β‐PCKO mice exhibited glucose intolerance with impaired glucose‐stimulated insulin secretion (GSIS) under either a standard chow (STC) diet or prolonged high‐fat diet feeding. β‐cell mass was significantly reduced in β‐PCKO mice, along with increased β‐cell senescence, decreased β‐cell proliferation, and unchanged β‐cell apoptosis. Peripheral insulin sensitivity and body weight were similar between β‐PCKO mice and WT littermates, indicating that PC is essential for glucose homeostasis by maintaining functional β‐cell mass. To date, the effect of β‐cell senescence on GSIS remains controversial. Studies have reported conflicting data, with some claiming improvement [8, 9] and others finding a decline [10] in GSIS under senescent state of β‐cells. Besides, the effects of aging on insulin secretion in humans and rodents are largely different [11]. Therefore, further investigation is warranted to clarify the role of β‐cell aging and/or senescence in insulin secretion.

Next, in order to gain a comprehensive understanding of PC on β‐cell functions, the researchers conducted RNA sequencing (RNAseq) in the isolated pancreatic islets of 12‐week‐old β‐PCKO mice and WT mice fed with STC. KEGG pathway analysis identified *p53* signaling as the top‐enriched pathway in β‐PCKO islets. Although best known for its activity as a tumor suppressor, *p53* signaling pathway was also reported to control cell proliferation, apoptosis, and senescence [12], which might contribute to the reduction of β‐cell mass in β‐PCKO mice. Transcriptomic analysis revealed that p53‐related senescence was activated in the β‐PCKO islets. QPCR analysis confirmed the senescent gene *Cdkn1a*, and the cell cycle genes such as *Ccnb1* and *Cdk2* but not the apoptotic genes such as *Caspase9*, *Bid*, and *Fas*, were influenced by deletion of *Pc* in the pancreatic islets. To examine whether PC controls p53 pathway in β‐cell line, the researchers silenced *Pc* expression in INS‐1E cells using siRNA. Knockdown of PC significantly increased p53 and p21 protein levels. The researchers then validated that *Pc* silencing induces senescent responses in β‐cells under hyperglycemia condition using multiple experimental approaches, including (a) senescence‐associated secretory phenotype (SASP), (b) β‐galactosidase (SA‐β‐gal) activity, (c) cell morphological changes (i.e., enlarged cell size and disrupted nuclear membrane), (d) DNA damage, and (e) cell cycle arrest. Results indicated that PC knockdown exacerbates β‐cell senescence in a p53‐dependent manner. Meanwhile, overexpression of PC inhibited hyperglycemia‐ and aging‐induced p53‐related senescence in human and mouse islets as well as INS‐1E β‐cells.

As a central player in glucose‐induced β‐cell failure [13], p53 is also critical for the β‐cell senescence progress [14]. Mdm2 is the predominant regulator of p53 [15]. A key observation of this study is that PC is able to bind with MDM2 and prevent its degradation via its MDM2 binding motif. Transcriptomic analysis identified *Mdm2* as the hub gene connecting to the p53 pathways. Given that MDM2 protein expression was lower in the islets of β‐PCKO mice, while no difference was observed in *Mdm2* mRNA levels. The researchers reasoned that PC should control MDM2 expression at the post‐translational level. Mechanistically, PC interacted with MDM2 to prevent its degradation via the MDM2 binding motif, which in turn restricts the p53‐dependent senescent program in β‐cells. Notably, the authors showed that PC protein levels and *PC* gene expression are decreased in β‐cells from aged and diabetic subjects, and that PC overexpression rescued GSIS in diabetic patients. These results not only further support that this enzyme is required for the correct function of β‐cells in vivo but also imply PC as a promising therapeutic target for diabetes.

Accumulating studies indicated that β‐cell aging and cellular senescence contribute to type 2 diabetes mellitus (T2DM) [16], but the underlying mechanisms are yet to be fully understood. In our study, we reported that PC performs anti‐senescent function in addition to its catalytic activity. PC involves in GSIS via the MDM2–p53–PC axis and plays anti‐senescence function via the PC–MDM2–p53 axis, highlighting the vital role of PC in diabetes therapy and/or prevention. Importantly, the researchers proposed that PC exerts its anti‐senescent function by protein binding, which identified PC as a moonlighting enzyme. Moonlighting protein refers to the observation that a single protein can have two or even more functions. Moonlighting by enzymes is an important example of the protein “multi‐tasking” [17]. A thorough investigation of the underlying functions of metabolic enzymes will contribute to a better understanding of metabolic disease.

In summary, Yang et al. uncovered PC as a multi‐functional metabolic enzyme that plays important roles in GSIS and β‐cell senescence. PC prevents β‐cell senescence by stabilizing MDM2 protein, thereby restricting p53‐mediated senescent program in both rodent and human β‐cells. PC is essential for maintaining healthy β‐cell functions in diabetic and aging conditions. Considering that this article only included hyperglycemia‐induced senescence for mechanism study, further investigation is warranted to determine whether the PC–MDM2–p53 axis is also involved in senescence induced by other insults such as DNA damage, cytokines, and toxic lipids. Overall, pharmacological targeting of PC could represent an exciting preventive and alleviating strategy for T2DM.

## Conflicts of Interest

The authors declare no conflicts of interest.
